# Concomitant responses of free-living nitrogen fixation and diazotrophic community composition to soil moisture in two soils

**DOI:** 10.3389/fmicb.2026.1915701

**Published:** 2026-08-26

**Authors:** Qian Zhao, Qingqing He, Jipeng Wang

**Affiliations:** 1 School of Emergency Management, Xihua University, Chengdu, China; 2 School of Ecology, Hainan University, Haikou, China; 3 Mountain Ecological Restoration and Biodiversity Conservation Key Laboratory of Sichuan Province, Chengdu Institute of Biology, Chinese Academy of Sciences, Chengdu, China

**Keywords:** asymbiotic nitrogen fixation, climate change, diazotrophic biofertilizer, *nifH* gene, nitrogen cycle, soil diazotrophs

## Abstract

Free-living nitrogen fixation (FLNF) in soil is an important but still poorly constrained source of nitrogen input to terrestrial ecosystems. Although moisture is widely recognized as a major driver of soil FLNF, mechanisms underlying the moisture-related variation in FLNF remain unclear. Here, we investigated the responses of soil FLNF to a controlled moisture gradient ranging from 20 to 100% of water holding capacity (WHC) in a cropland soil and a forest soil. Our results showed that potential FLNF rates increased non-linearly with increasing soil moisture, rising by more than two orders of magnitude at 60% (cropland soil) and 80% (forest soil) WHC relative to drier treatments. In contrast to FLNF, soil respiration declined at the wetter end of the gradient, a pattern consistent with increasing aeration constraints. Together, the divergent moisture dependences of FLNF and respiration support the hypothesis that reduced oxygen inhibition on nitrogenase may have contributed to the high FLNF under wet conditions. Furthermore, moisture-related changes in FLNF coincided with shifts in both total (DNA-based) and active (RNA-based) diazotrophic community composition. In particular, *Azotobacter* (cropland soil) and *Paenibacillus* (forest soil) increased in relative abundance and activity under wetter conditions and were positively associated with FLNF rates, indicating that changes in these taxa may be linked to the moisture-related variation in FLNF. Overall, our study reveals pronounced moisture sensitivity of potential FLNF under controlled conditions and highlights soil aeration and diazotrophic community composition as testable mechanisms. Further validation across soils and sites under field conditions is needed before these findings can be extrapolated to ecosystem-scale nitrogen fixation or biofertilizer applications.

## 1 Introduction

Free-living nitrogen fixation (FLNF) is performed by diazotrophic microbes that fix atmospheric nitrogen (N) without forming obligate symbiotic associations with plants (Smercina et al., 2019b). Unlike symbiotic N fixation, FLNF is not confined to root nodules and occurs across diverse habitats, including forest canopies, soils, litter, dead wood, ground

mosses, biological soil crusts, and epiphytic lichens (Cleveland et al., 2022; Reed et al., 2011). Among these niches, soils represent a major reservoir of FLNF, contributing approximately 26% of total biological N fixation (BNF; the sum of free-living and symbiotic N fixation) in natural terrestrial ecosystems (Reis Ely et al., 2025). Despite its substantial contribution to ecosystem N inputs, soil FLNF has yet not been explicitly represented in most Earth system models (Reis Ely et al., 2025). In parallel, efforts to harness diazotrophs as biofertilizers often fall short because field N fixation rates are typically low and highly variable (Chakraborty et al., 2024). Both modeling uncertainty and limited agronomic efficacy stem, in part, from an incomplete understanding of the environmental controls on soil FLNF (Chakraborty et al., 2024; Cleveland et al., 2022).

Soil FLNF is governed by interacting biophysical drivers (e.g., temperature and moisture) and biogeochemical constraints (e.g., energy supply, oxygen availability, and nutrients) (Cleveland et al., 2022; Smercina et al., 2019b). Among these factors, soil moisture is widely viewed as a primary regulator via multiple pathways. First, soil moisture can directly constrain diazotroph physiology by altering cellular hydration and osmotic stress, potentially generating a unimodal response of FLNF to increasing water availability (Cleveland et al., 2022). Under extremely dry conditions, FLNF may remain negligible even when substrates and nutrients are sufficient (Gundale et al., 2012; Kroeckel and Stolp, 1984; Roley et al., 2018). Second, soil moisture indirectly regulates FLNF by controlling oxygen (O_2_) availability through its effects on soil pore connectivity and gas diffusion (Davidson, 1993; Moldrup et al., 2004). Because nitrogenase is irreversibly inactivated by O_2_, drier soils with higher gas diffusivity may impose greater energetic costs for nitrogenase protection (e.g., respiratory protection and diffusion barriers), thereby reducing N fixation rates (Dixon and Kahn, 2004; Inomura et al., 2017). Third, these moisture-driven physiological and oxygen constraints may act as selective pressures on diazotrophic communities, altering their composition and activity. Growing evidence suggests that structural shifts within diazotrophic communities are closely linked to variation in FLNF rates (Hsu and Buckley, 2009; Hu et al., 2024; Wang et al., 2023; Zhao et al., 2024). However, the abovementioned mechanistic linkages remain largely inferential. In particular, direct empirical demonstrations that moisture regulates FLNF through moisture-driven community change remain limited. Filling this gap is increasingly urgent as climate change intensifies droughts and extreme precipitation events (IPCC, 2023; Trenberth et al., 2014).

The objectives of this study were to quantify the response of FLNF to soil moisture and to evaluate the processes potentially associated with this response. To this end, we conducted a controlled moisture manipulation experiment using a cropland soil and a forest soil. The soils were incubated across a moisture gradient ranging from 20–100% of water holding capacity (WHC). We quantified potential FLNF and examined its relationships with soil respiration (used as an indicator of overall microbial metabolic activity and potential aeration limitation), as well as with the composition of both total (DNA-based) and active (RNA-based) diazotrophic communities. We hypothesized that, within the studied moisture range, (1) potential FLNF would increase with soil moisture, consistent with the alleviation of water stress and possibly reduced oxygen-related constraints on nitrogenase; and (2) moisture-related increases in FLNF would coincide with compositional shifts in diazotrophic communities due to potential differences among taxa in their responses to soil moisture.

## 2 Materials and methods

### 2.1 Study site and sample collection

Soils were collected from the Yanting Station of the Chinese Ecosystem Research Network (CERN) in the central Sichuan Basin, China (31°16′N, 105°27′E; 360–600 m a.s.l.). The region has a subtropical monsoon climate with a mean annual temperature of 17.5 °C and mean annual precipitation of 826 mm. The forest site is a mixed plantation of *Cupressus funebris* and *Alnus cremastogyne*. The cropland site follows a winter wheat–summer maize rotation and receives annual fertilization of 750 kg ha^−1^ yr^−1^ NH_4_HCO_3_, 600 kg ha^−1^ yr^−1^ Ca(H_2_PO_4_)_2_, and 75 kg ha^−1^ yr^−1^ KCl.

At each land use type, surface mineral soils (0–10 cm) were collected and homogenized to form one composite sample, passed through a 2-mm sieve to remove roots and residues, and stored at 4 °C prior to incubation. Both soils are classified as Cambisols with pH values of 8.2. The forest soil has higher soil organic carbon (SOC) content than the cropland soil (5.3% and 2.6%, respectively). Detailed information of soil properties including pH, bulk density, SOC, total nitrogen (TN), extractable inorganic N (EIN), extractable inorganic P (EIP), soil water content (SWC), and WHC are reported in Supplementary Table S1.

### 2.2 Moisture manipulation experiment

A controlled moisture manipulation experiment was conducted following Seuss et al. (2022) with minor modifications. Composite cropland and forest soils were processed independently throughout the experiment. Each composite soil was air-dried at room temperature to < 10% of its water-holding capacity (WHC), thoroughly homogenized, and adjusted to five moisture treatments with final target levels of 20%, 40%, 60%, 80%, and 100% WHC. For each soil and target moisture level, 24 independent soil microcosms were established, each containing 10 g dry-mass-equivalent soil in a separate polyethylene bag. Before pre-incubation, deionized water was added individually to each microcosm to adjust soil moisture to 10%, 30%, 50%, 70%, or 90% WHC for the treatments subsequently adjusted to 20%, 40%, 60%, 80%, or 100% WHC, respectively. All microcosms were pre-incubated at 25 °C for 3 days in polyethylene bags under aerobic conditions. After pre-incubation, a C cocktail solution was added individually to each microcosm to simultaneously (i) achieve final moisture levels of 20%, 40%, 60%, 80%, and 100% WHC, and (ii) provide 2 mg C g^−1^ dry soil to minimize C limitation of FLNF (Chiewattanakul et al., 2022; Liu et al., 2022; Smercina et al., 2019a). The C cocktail consisted of citric acid (CA), malic acid (MA), glucose (Glc), and sucrose (Suc), with each compound contributing an equal amount of added C (Smercina et al., 2019a; Zhao et al., 2024).

Following C addition, all microcosms were incubated at 25 °C under aerobic conditions. Destructive sampling was conducted on Days 1, 3, 5, 7, 10, and 14 after C addition. For each soil and each time point, four replicate subsamples per moisture treatment were removed for measurements. Specifically, 5 g soil (dry mass equivalent) was used for FLNF assays and 0.2 g for soil respiration measurements. For molecular analyses, an additional 2 g soil was collected each microcosm harvested on Day 3, flash-frozen in sterile centrifuge tubes in liquid N_2_, and stored at −80 °C until nucleic acid extraction. During incubation, bags were weighed daily and deionized water was added as needed to compensate for water loss and maintain target moisture levels. Moisture effects were evaluated separately within the cropland and forest soils, and no statistical comparison between the two soil sources was intended.

### 2.3 Measurement of potential FLNF rate

Potential FLNF rate was quantified using the acetylene reduction assay (ARA) following Wang et al. (2023). Briefly, 5.0 g soil (dry mass equivalent) was placed in a 65-mL gas-tight Erlenmeyer flask and sealed with a butyl rubber stopper. Acetylene (C_2_H_2_) was injected to replace 10% (v/v) of the headspace volume, and the flasks were then incubated at 25 °C for 24 h. Two controls were included in parallel: (i) soil-only flasks to correct for endogenous ethylene (C_2_H_4_) production, and (ii) C_2_H_2_-only flasks to correct for potential C_2_H_4_ contamination in the C_2_H_2_ gas. After incubation, headspace C_2_H_4_ concentrations was determined using a gas chromatograph (Shimadzu GC-2010 Pro) equipped with a Porapak N column and flame ionization detector. The ARA-based FLNF rate (μg C_2_H_4_ g^−1^ soil d^−1^) was calculated as:


$$\begin{array}{l} FLNF\;rate=\;\frac{C\times 10^{-3}\times V_{h}\times M}{V_{m}\times t\times m} \end{array}$$

where *C* is the C_2_H_4_ concentration (ppmv), *V*_*h*_ is the headspace volume (mL), *M* is the molecular mass of C_2_H_4_ (28 g mol^−1^), *V*_*m*_ is the molar gas volume at 25 °C and 101 kPa (24.5 L mol^−1^), *t* is the incubation time (d), and *m* is the dry soil mass (g). The ARA employed in this study quantifies nitrogenase activity under C-replete laboratory conditions and therefore represents potential rather than *in situ* N fixation rates. This experimental design allows the influence of soil moisture to be evaluated independently of C limitation. In a previous study, we validated ARA-derived estimates against 15N_2_ incorporation measurements and observed a strong linear relationship between the two methods (Zhao et al., 2024; *R*^2^ = 0.91 and 0.96, respectively for the cropland and forest soils, *P* < 0.001 for both), supporting the reliability of ARA as a proxy for relative variation in FLNF. Although absolute rates may differ between methods, ARA reliably captures treatment-induced differences in nitrogenase activity

### 2.4 Soil respiration rate

Soil respiration was determined using the alkali absorption method (Alef, 1995) with slight modifications. Briefly, 0.20 g soil (dry mass equivalent) was placed in a 2-mL centrifuge tube and incubated inside a sealed 50-mL conical flask containing 4 mL of 0.05 M NaOH to trap evolved CO_2_. Blank controls containing NaOH without soil were included to correct for background CO_2_. Flasks were incubated at 25 °C for 24 h. After incubation, the NaOH solution was precipitated with 1.25 mL of 0.5 M BaCl_2_, and phenolphthalein (0.1 mL) was added as an indicator. The remaining NaOH was titrated with standardized 0.005 M HCl until the endpoint was reached. Soil respiration rate (mg CO_2_ g^−1^ soil d^−1^) was calculated as:


$$\begin{array}{l} Respiration\;rate=\;\frac{a\times \left(V_{0}-V\right)\times \;1.1}{t\times m} \end{array}$$

where *a* is the correction factor for the exact HCl concentration, *V*_0_ and *V* are the volumes (mL) of HCl used for the blank and sample, respectively, *1.1* is the conversion factor (1 mL of 0.05 M NaOH corresponds to 1.1 mg CO_2_), *t* is incubation time (d), and *m* is the dry soil mass (g).

### 2.5 Nucleic acid extraction, cDNA synthesis and nifH amplicon sequencing

Soil DNA and RNA were extracted from Day-3 samples using the FastDNA SPIN Kit for Soil (MP Biomedicals, Unites States) and a Soil RNA Extraction Kit (Majorbio, China), respectively, following manufacturer protocols. DNA/RNA quantity and quality were assessed using a NanoDrop-2000 spectrophotometer and 1% agarose gel electrophoresis. Residual DNA in RNA extracts was removed with Recombinant DNase I (TaKaRa Bio), and cDNA was synthesized using HiScript II Reverse Transcriptase (Vazyme Biotech, China).

The *nifH* gene was amplified from DNA and cDNA using primers nifH-F and nifH-R (Wang et al., 2023) with sample-specific barcodes. PCRs (20 μL) contained 4 μL 5 × FastPfu buffer, 2 μL 2.5 mM dNTPs, 0.8 μL of each primer (5 μM), 0.4 μL FastPfu polymerase, 0.2 μL BSA, ~10 ng template, and nuclease-free water. Thermal cycling: 95 °C 3 min; 40 cycles of 95 °C 5 s, 60 °C 30 s, 72 °C 45 s; final extension 72 °C 10 min. Triplicate PCRs per sample were pooled, purified (TaKaRa Agarose Gel DNA purification kit), and sequenced on an Illumina NextSeq 2000 platform.

Raw reads were quality-filtered using fastp (v0.19.6) and merged with FLASH (v1.2.11). Reads with Q < 20 or ambiguous bases were removed. FrameBot in the FunGene pipeline was used to correct frameshifts; sequences were translated, screened to remove stop codons and chimeras, and retained as nifH protein-coding sequences. High-quality sequences were clustered into OTUs at 97% identity using USEARCH11. Representative OTU sequences were taxonomically assigned with the RDP Classifier against the FunGene nifH database at a confidence threshold of 0.7. Sequencing generated 6,159,222 high-quality nifH sequences for DNA samples (30,223–66,265 per sample) and 3,112,973 for cDNA samples (50,872–82,091 per sample).

### 2.6 Statistical analyses

All statistical analyses were performed in R (v4.2.1) and visualized using ggplot2. To evaluate the effects of moisture and sampling time on FLNF and respiration rates, a two-way factorial linear model with soil moisture, sampling time, and their interaction as fixed effects was fitted using ordinary least squares. Overall effects were evaluated using HC3 heteroscedasticity-robust Type III Wald F tests. Because FLNF rate contained zero observations and was therefore analyzed as log(FLNF + c), where c was defined a priori as one-half of the minimum positive FLNF value. When the moisture × sampling-time interaction was significant, inference focused on simple effects in which moisture levels were compared within each sampling time, and sampling times were compared within each moisture level. Pairwise comparisons were adjusted using the Holm procedure.

Within each soil type, time-integrated FLNF rate between days 1 and 14 was calculated for each moisture treatment using trapezoidal integration of the treatment-level mean rates measured at 1, 3, 5, 7, 10, and 14 d (Cumulative FLNF rate, μg C_2_H_4_ g^−1^ soil):


$$\begin{array}{l} Cumulative\;FLNF\;rate=\sum_{i=1}^{5}\frac{t_{i\;+\;1}\;-\;t_{i}}{2}\left(Rate_{j}+Rate_{j+1}\right) \end{array}$$

where *i* indexes sampling times, $Rate_{j}$ is the mean FLNF rate at time *t*_*i*_. Uncertainty was quantified using 10,000 stratified bootstrap resamples conducted independently within each sampling time, from which percentile-based 95% confidence intervals were calculated. Differences in Cumulative FLNF rate among moisture treatments were evaluated separately for each soil using 9,999 stratified permutations, with moisture labels permuted within sampling times. Pairwise permutation *P*-values were adjusted using the Holm procedure. The same method was used to calculate the cumulative respiration rate and compare its differences among moisture treatments.

The relationship between soil moisture and cumulative FLNF activity was evaluated using seven candidate models, including linear, exponential, logarithmic, quadratic polynomial, power, logistic, and Gaussian peak functions. Linear models were fitted using ordinary least squares, whereas non-linear logistic and Gaussian models were fitted using a bounded limited-memory Broyden-Fletcher-Goldfarb-Shanno optimization algorithm (L-BFGS-B) with multiple starting values to reduce sensitivity to initial parameter estimates and avoid convergence to local optima. Model performance was assessed using leave-one-moisture-level-out cross-validation (LOOCV). Prediction performance was evaluated using both raw-scale RMSE and RMSE calculated after inverse hyperbolic sine transformation (asinh), which allows robust evaluation of zero and slightly negative predictions. Errors were normalized against a null model to quantify the relative improvement of each candidate model. The final model selection score combined normalized prediction errors from both scales, negative prediction penalties, prediction instability penalties, shape constraints for peak models, and model complexity penalties, and lower scores indicate better model performance.

Principal coordinates analysis (PCoA) was used to analyze the moisture-related shifts in diazotrophic community composition based on Bray-Curtis distances at the genus level. Moisture effects were tested using PERMANOVA (999 permutations). The “envfit” function in the “vegan” package was used to fit the FLNF rate measured on Day 3 onto the PCoA ordination space, which quantifies the relationship between the FLNF rate and the community composition, providing *R*^2^ and associated permutation test *P*-values (999 permutations) to assess the significance of the relationship.

## 3 Results

### 3.1 Potential FLNF rate increased with soil moisture in a non-linear manner

**Figure 1:**
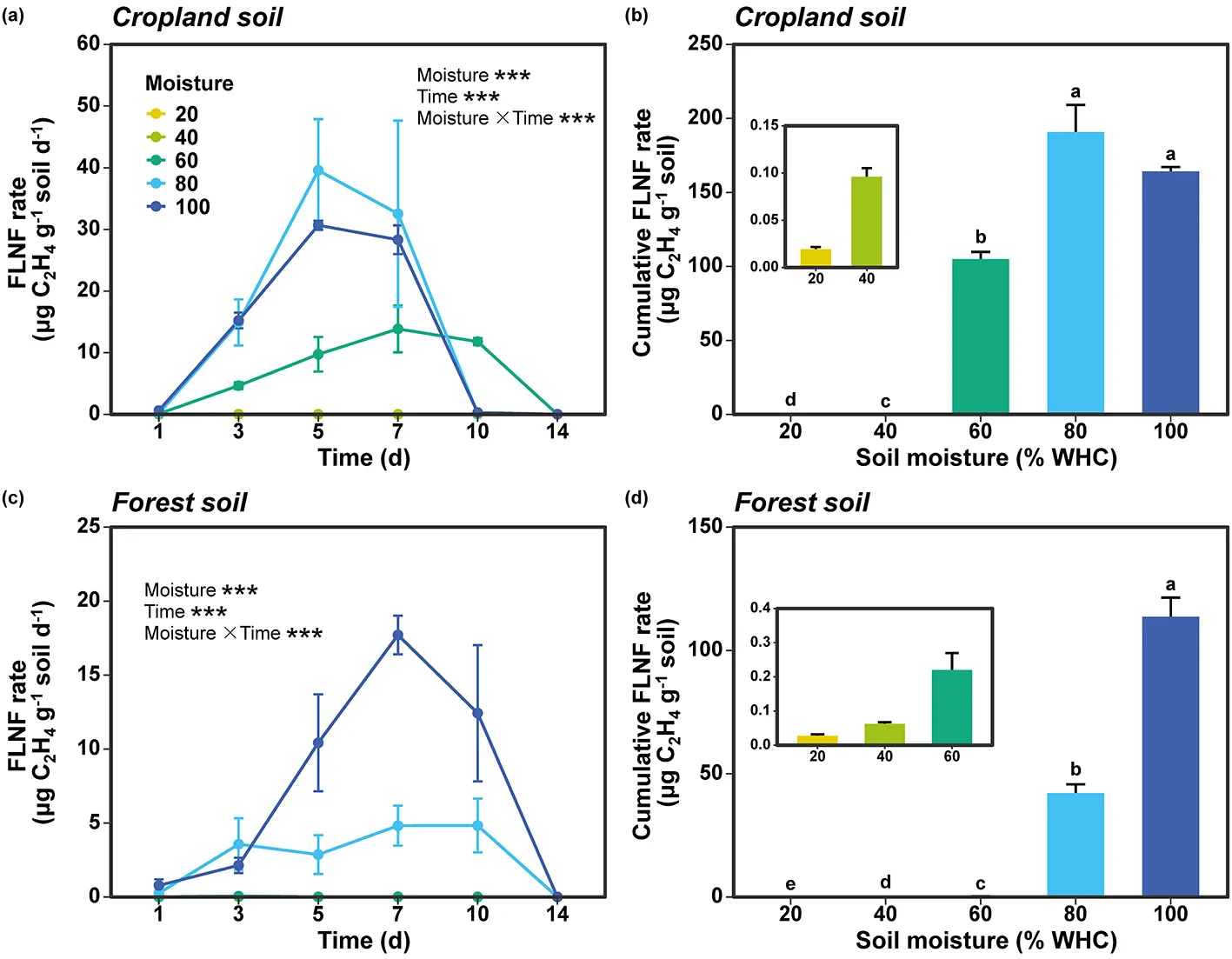
Effects of soil moisture (%water holding capacity, WHC) on the free-ling nitrogen fixation (FLNF) rates in cropland **(a, b)** and forest **(c, d)** soils. **(a)** and **(c)** display the measured FLNF rate under different soil moisture levels on Days 1, 3, 5, 7, 10, and 14, respectively (mean ± standard deviation, *n* = 4). Statistical results for soil moisture, sampling time, and their interaction are embedded within each panel, with *** indicating *P* < 0.001. **(b)** and **(d)** present the cumulative FLNF rate integrated across Days 1–14 (mean + standard deviation, *n* = 4), and different lowercase letters indicate significant differences between different soil moisture levels.

Potential FLNF rate (ARA-based C_2_H_4_ production per day) significantly varied among soil moisture levels and sampling time, and the interaction effects of moisture and sampling time were also significant (Figures 1a, c; Supplementary Tables S2–S4). Cumulative FLNF rate across 1 to 14 days increased markedly with increasing soil moisture (expressed as %WHC) in both cropland and forest soils (Figures 1b, d). Specifically, cumulative FLNF rates remained low (< 0.10 μg C_2_H_4_ g^−1^ soil) at low moisture ( ≤ 40% WHC), while it increased sharply at higher moistures, rising by at least two orders of magnitude once moisture reached 60% WHC in the cropland soil and 80% WHC in the forest soil (Figures 1b, d).

Together, the relationships between soil moisture and cumulative FLNF rate were best described by non-linear models, consistent with the steepest increases occurring at 60% or 80% WHC (Figure 1; Supplementary Table S5). For the cropland soil, the logistic model provided the strongest predictive performance, achieving the best composite selection score (0.181), while the relationship for forest soil was best represented by a power model (selection score = 0.419) (Supplementary Table S5).

### 3.2 Soil respiration showed a distinct moisture dependence relative to FLNF

**Figure 2:**
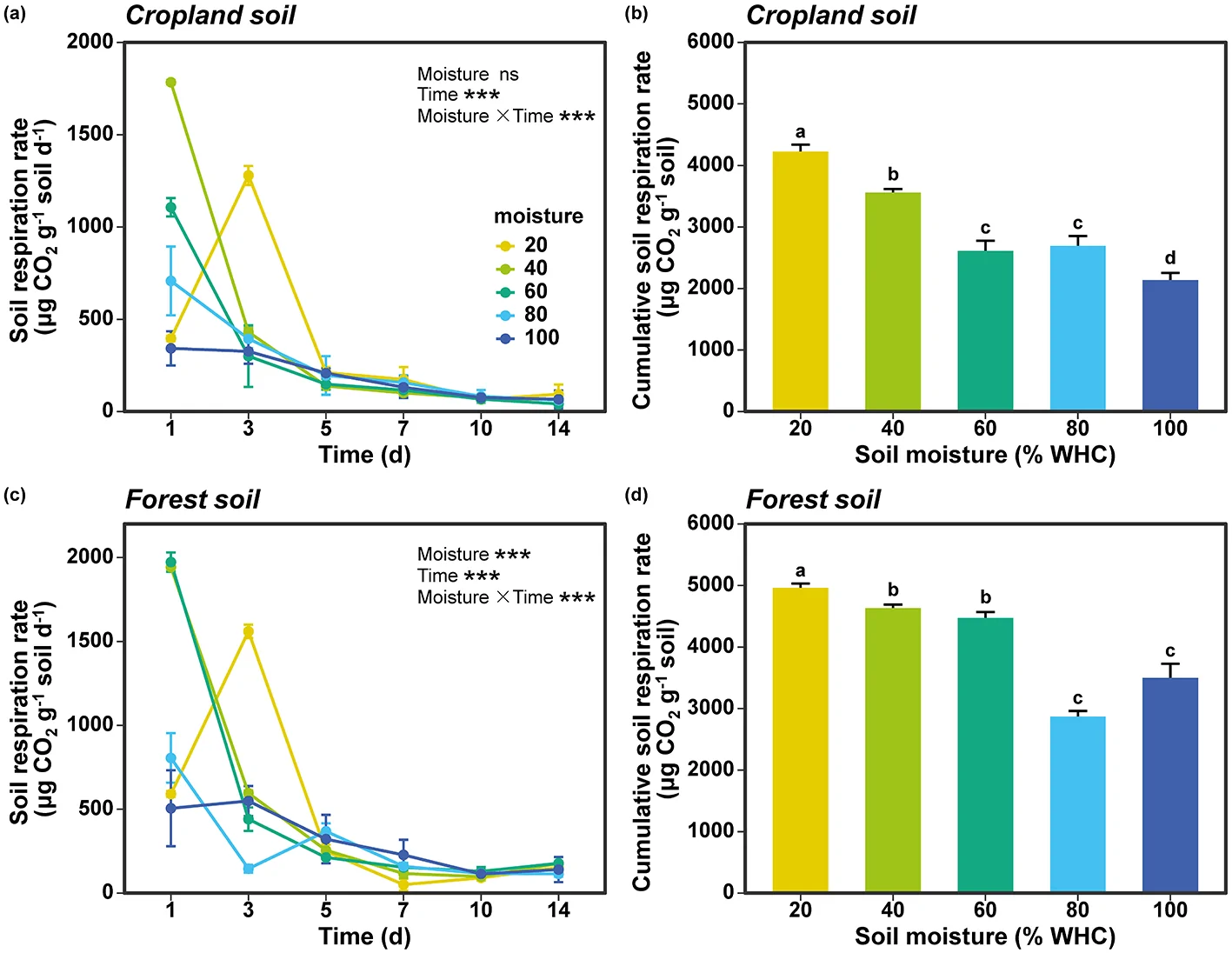
Effects of soil moisture (%water holding capacity, WHC) on the respiration rates in cropland **(a, b)** and forest **(c, d)** soils. **(a)** and **(c)** display the measured respiration rate under different soil moisture levels on Days 1, 3, 5, 7, 10, and 14, respectively (mean ± standard deviation, *n* = 4). Statistical results for soil moisture, sampling time, and their interaction are embedded within each panel, with *** indicating *P* < 0.001 and ns indicating not significant at *P* = 0.05 level. **(b)** and **(d)** present the cumulative respiration rate integrated across Days 1–14 (mean + standard deviation, *n* = 4), and different lowercase letters indicate significant differences between different soil moisture levels.

Soil respiration rate was under the interacted influences of soil moisture and sampling time (Figure 2a, c; Supplementary Tables S2–S4). Cumulative respiration rate declined with increasing soil moisture in both soils (Figures 2b, d). In cropland soil, respiration was highest at 20% WHC (4230 μg CO_2_ g^−1^ soil) and decreased progressively to its minimum at 100% WHC (Figure 2b). Similarly, forest soil showed relatively high respiration at 40–60% WHC (>4470 μg CO_2_ g^−1^ soil), whereas respiration at 80–100% WHC declined to < 3,000 μg CO_2_ g^−1^ soil (Figure 2d). These results is consistent with the hypothesis that higher moisture levels constrained overall aerobic metabolic activity and aeration during incubation, which coincided with the higher rates of FLNF observed in Figure 1.

### 3.3 Moisture-related shifts in diazotrophic communities coincided with the increase in FLNF rate

**Figure 3:**
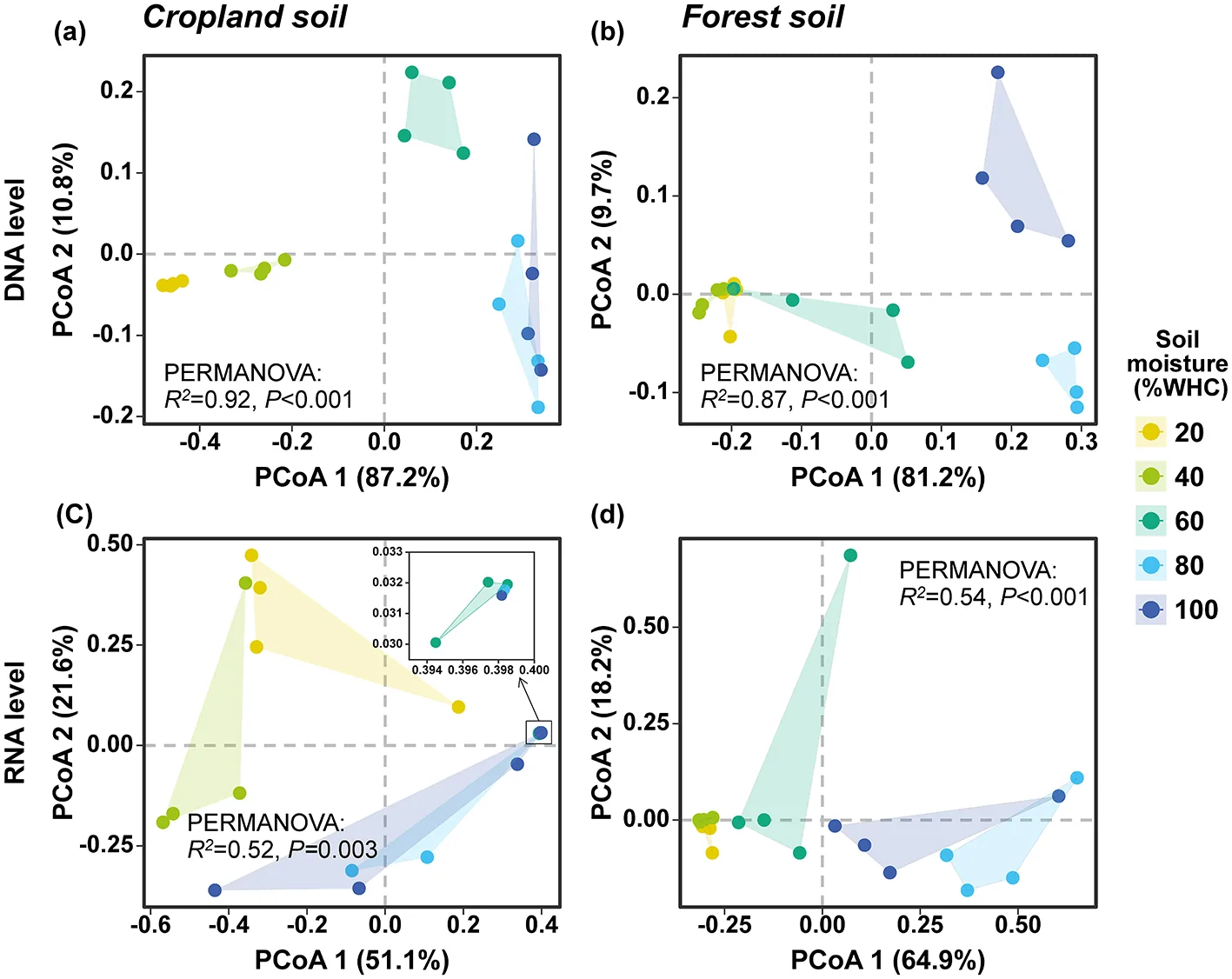
Principal coordinates analysis (PCoA) of the diazotrophic community composition at the genus level in the cropland **(a, c)** and forest **(b, d)** soils under different soil moisture (%water holding capacity, WHC) on Day 3. The results of permutational multivariate analysis of variance (PERMANOVA) are shown in each panel.

**Figure 4:**
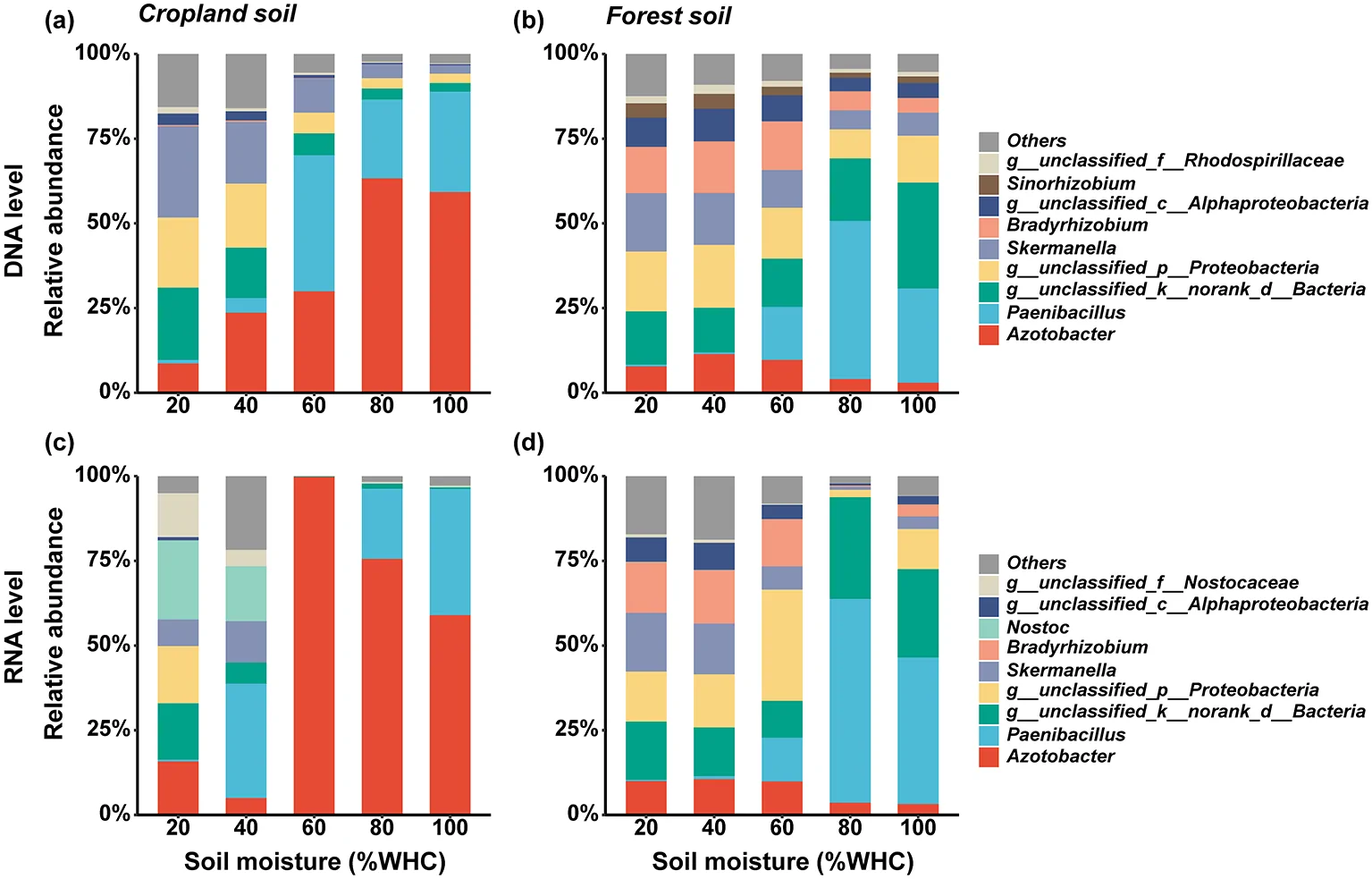
Composition of diazotrophic community at the genus level in the cropland **(a, c)** and forest **(b, d)** soils under different soil moisture (%water holding capacity, WHC) on Day 3. Mean values of 4 replicates are shown.

On Day 3, both DNA- and RNA-based *nifH* amplicons revealed clear moisture-related separation of diazotrophic community composition at the genus level (Figures 3, 4). PCoA showed that communities clustered by moisture treatment and were significantly differentiated among %WHC levels in both cropland and forest soils (Figure 3). Notably, community compositions shifted most strongly at the same moisture levels where FLNF began to increase sharply (i.e., 60% WHC in cropland soil and 80% WHC in forest soil), particularly along the PCoA axis 1 (Figure 3). In cropland soil, the relative abundance of *Azotobacter* increased at higher moisture levels (*P* < 0.01 and *P* = 0.055 at DNA and RNA levels, respectively; Figures 4a, c and Supplementary Figures S1, S2. In forest soil, *Paenibacillus* increased with moisture and dominated the community at 80% WHC, with consistent patterns at both DNA and RNA levels (*P* < 0.01 and *P* < 0.05, respectively; Figures 4b, d; Supplementary Figures S3, S4).

**Figure 5:**
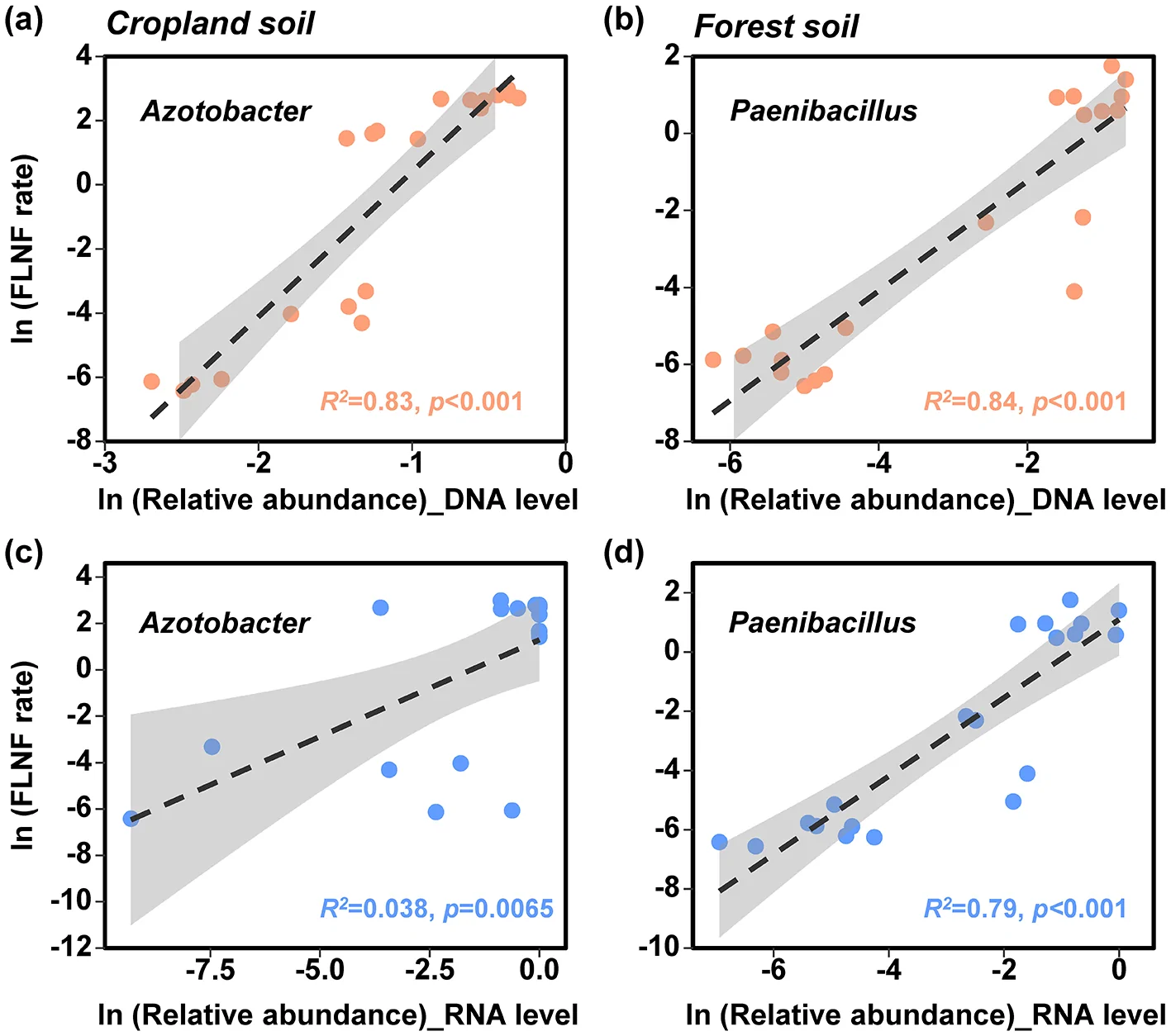
Relationship between the relative abundances of *Azotobacter* in the cropland soil **(a, c)** and *Paenibacillus* in the forest soil **(b, d)** with FLNF rate under different soil moisture levels (%water holding capacity, WHC) on Day 3. Data were ln-transformed before analysis.

Changes in diazotrophic community composition were significantly associated with variation in FLNF across moisture treatments (Supplementary Tables S6, S7), indicating that moisture-driven restructuring of the diazotrophic community may track changes in N fixation activity. In addition, at both DNA and RNA levels, the relative abundances of *Azotobacter* in cropland soil were positively related to the FLNF rate measured on Day 3 (Figures 5a, c; Supplementary Table S7), and *Paenibacillus* abundance was positively related to the FLNF rate in forest soil measured on Day 3 (Figure 5b, d; Supplementary Table S7). Collectively, these results support a close relationship between moisture-induced community shifts and enhanced FLNF at high soil moisture.

## 4 Discussion

### 4.1 Increased potential FLNF rate under high soil moisture

Consistent with our first hypothesis, potential FLNF increased strongly with increasing soil moisture in both cropland and forest soils (Figure 1). This positive moisture dependence under controlled conditions aligns with field observations showing enhanced soil FLNF in wet soils (Rousk et al., 2018; Zhang et al., 2020) or following rainfall pulses (Vizza et al., 2024). One possible mechanism underlying this response is the effect of soil moisture on O_2_ availability. As soil moisture increases, water occupies a greater proportion of the soil pore space, reducing air-filled porosity, pore connectivity, and gas diffusivity. These changes can restrict O_2_ transport and promote the development of relatively O_2_-limited microsites (Davidson, 1993; Moldrup et al., 2004). Because nitrogenase is highly sensitive to O_2_, reduced O_2_ availability may alleviate oxygen inhibition of nitrogenase and decrease the energetic investment required by diazotrophs for protective mechanisms, such as respiratory protection and diffusion barriers (Dixon and Kahn, 2004; Inomura et al., 2017). Consequently, wetter conditions may provide a more favorable oxygen condition for N fixation. The observed response of soil respiration provides indirect support for this interpretation. In contrast to FLNF, respiration declined at the wetter end of the moisture gradient, particularly at 60–100% WHC in the cropland soil and 80–100% WHC in the forest soil (Figure 2). Reduced CO_2_ production under high soil moisture is commonly associated with lower gas diffusivity, restricted O_2_ supply, and suppression of bulk aerobic microbial metabolism (Skopp et al., 1990). Thus, the concurrent decline in respiration and increase in FLNF is consistent with a moisture-induced transition toward stronger aeration limitation, under which O_2_-sensitive nitrogenase activity may be favored relative to aerobic heterotrophic metabolism. Moreover, higher soil moisture may enhance the diffusion of dissolved C and other substrates within soil water films (Davidson, 1993; Seuss et al., 2022). The increased substrate diffusion, together with the suppression of bulk aerobic metabolism, suggests that wetter conditions could reduce the resource competition between diazotrophs and other microbes. Collectively, these mechanisms may disproportionately benefit diazotrophs, a low-abundance functional guild which is often oxygen-sensitive (Smercina et al., 2019a) and can be relatively weak competitors for soil substrates (Jones et al., 2003; Schimel and Schaeffer, 2012; Zhao et al., 2024). On the other hand, respiration remained comparatively high at 20–40% WHC (Figure 2), suggesting that low moisture did not strongly constrain whole-community metabolism under our incubation conditions. This pattern provides limited support for the alternative explanation in our first hypothesis that increasing moisture stimulated FLNF directly by alleviating dehydration or osmotic stress. However, this inference should be made cautiously because diazotrophs may have different water-stress sensitivities and activity thresholds than the bulk microbial community.

A key feature of our results is the non-linear moisture response of FLNF, with an abrupt increase (~2 orders of magnitude) between 40% and 60% WHC in cropland soils and between 60% and 80% WHC in forest soils (Figure 1; Supplementary Table S5). Such thresholds are consistent with the idea that soil processes shift rapidly once air-filled porosity and gas diffusivity cross a critical range when O_2_ diffusion becomes largely restricted (Davidson, 1993; Moldrup et al., 2004). In our experiment, these FLNF surges coincided with the onset of depressed respiration at high moisture (Figure 2), supporting a moisture-driven transition toward stronger aeration constraints. The higher apparent threshold in forest soil likely reflects differences in physical structure and water retention (e.g., lower bulk density and higher SOC relative to cropland; Supplementary Table S1), which may maintain greater pore connectivity and gas transport at a given %WHC. Ecologically, such non-linear moisture responses offer a mechanistic explanation for why soil FLNF is frequently characterized by spatial and temporal “hot spots” and “hot moments,” rather than varying smoothly across landscapes and seasons (Soper et al., 2021). Rainfall-driven pulses, for instance, can rapidly move soils across the critical moisture range and trigger episodic spikes N fixation pulses in soil (Vizza et al., 2024). Nonetheless, although such critical moisture range has also been reported in Chilean soils, their reported threshold (20% WHC) was apparently lower than those reported in our study (Seuss et al., 2022), likely reflecting differences in soil physical constraints, substrate availability, and microbial adaptation. Together, these observations argue for explicitly incorporating non-linear moisture responses into models rather than assuming linear moisture-FLNF relationships when predicting responses to altered precipitation regimes.

### 4.2 Moisture-related increases in FLNF coincided with diazotrophic composition shifts

Consistent with our second hypothesis, moisture-related increases in potential FLNF coincided with shifts in diazotrophic community composition. Across the moisture gradient, changes in diazotrophic β-diversity tracked variation in FLNF rates on Day 3 at the whole-community level (Figures 3, 4; Supplementary Table S6), indicating that compositional turnover occurred alongside changes in N fixation function. Beyond these community-wide patterns, we identified specific taxa whose relative abundances were positively associated with elevated FLNF under wetter conditions, most notably *Azotobacter* and *Paenibacillus* in cropland and forest soils, respectively (Figure 5; Supplementary Figure S1; Table S7). Importantly, these associations were consistent at both DNA- and RNA-based levels, implying that the taxon-specific moisture adaptation of soil diazotrophs may be related to variation in FLNF function. However, because these relationships are based on relative abundance data collected at a single time point, they should be interpreted as associations rather than evidence that particular taxa directly determined the observed FLNF response. Together, our findings are consistent with previous findings that environmental drivers regulate soil N fixation not only via immediate physiological constraints, but also by restructuring diazotrophic community composition (Hsu and Buckley, 2009; Hu et al., 2024; Zhao et al., 2024).

From an applied perspective, species belonging to the two diazotrophic genera most strongly associated with elevated FLNF in our study (*Azotobacter* and *Paenibacillus*) have been widely investigated as potential biofertilizers (Aasfar et al., 2021; Grady et al., 2016). Despite their documented N-fixing potential under laboratory conditions, their realized N contribution to plant N acquisition in the field is often modest and highly variable (Chakraborty et al., 2023). Our results raise the possibility that soil moisture, potentially through its effects on aeration and community assembly, may be one of several environmental factors associated with the activity of these taxa *in situ*. A recent study demonstrated that nanocoating strategies can encapsulate diazotrophic inoculants to enhance tolerance to desiccation and oxidative stress, thereby improving N-fixation efficiency (Liao et al., 2026). Although developed for phyllosphere applications, the same design principles could potentially be extended to soil systems, where fluctuating moisture and oxygen availability impose strong constraints on FLNF. Beyond material-based innovations, water management may also merit further investigation. Because wetting events can trigger transient FLNF episodes in soils (Vizza et al., 2024), synchronizing inoculation with irrigation or rainfall pulses, and maintaining soils within a moisture range that permits microaerobic niches, may increase the likelihood that introduced diazotrophs activate nitrogenase and contribute measurable N inputs to crops. Nevertheless, the present study did not directly evaluate inoculant performance or crop N uptake, and therefore does not provide evidence for specific biofertilizer or water-management recommendations.

### 4.3 Study limitations

Several limitations should be considered when interpreting the moisture responses of potential FLNF observed in this study:

(1) The proposed role of O_2_ availability is supported only indirectly. Soil respiration provides an integrated measure of whole-community metabolic activity and may reflect changes in aeration constraints under different moisture conditions. However, it does not directly quantify O_2_ concentration, gas diffusivity, or redox conditions within the microsites occupied by diazotrophs. Therefore, the concurrent decline in respiration and increase in FLNF under wetter conditions is consistent with the hypothesis that reduced aeration alleviated O_2_ inhibition of nitrogenase, but it does not demonstrate this mechanism conclusively.

(2) Changes in resource availability during incubation were not characterized. Although the initial extractable inorganic N and P concentrations were measured and all treatments received the same amount and composition of added C, the availability and temporal dynamics of these resources were not monitored because the amount of soil available from each destructively sampled microcosm was limited. Soil moisture can alter resource solubility, mineralization, diffusion, and microbial uptake, and these processes may influence both diazotrophic activity and competition between diazotrophs and the broader microbial community. Consequently, the effects attributed to moisture may partly include indirect effects mediated by changes in resource accessibility.

(3) The diazotrophic community was characterized only on Day 3. The DNA- and RNA-based community data therefore represent a single time point and cannot be assumed to describe diazotrophic community composition or activity throughout the entire incubation period. The observed associations between Day-3 FLNF rate and the relative abundances of dominant taxa indicate that community composition and FLNF covaried at that time point, but they do not establish that the identified taxa caused the FLNF response on that day. Moreover, relative abundance data cannot distinguish an absolute increase in a given taxon from a proportional increase caused by declines in other community members.

(4) The generality of the findings is limited by the experimental design. The experiment included one composite cropland soil and one composite forest soil collected from a single study site. The results therefore demonstrate moisture responses within these two soil systems but do not quantify variation among sites, soil types, or land-use categories. In addition, the uniform addition of a readily available C mixture was intended to reduce C limitation and isolate the response of potential FLNF to soil moisture. This C-replete incubation differs from field conditions, in which the quantity, composition, and spatial distribution of available C vary over time and interact with plant inputs, nutrient availability, and natural wetting-drying cycles. Accordingly, the rates measured here represent potential rather than *in situ* FLNF, and the observed moisture thresholds should not be directly extrapolated to ecosystem-scale N inputs.

(5) The limitations listed above also constrain the immediate application of our findings to diazotroph-based biofertilizers. Although the moisture-associated responses of genera such as *Azotobacter* and *Paenibacillus* provide testable hypotheses regarding environmental conditions that may favor diazotrophic activity, they do not demonstrate that manipulating soil moisture would improve inoculant performance under field conditions.

## 5 Conclusions

Overall, we found that soil moisture exerted a strong, non-linear effect on potential FLNF across a controlled gradient from 20% to 100% WHC in two soils collected from the cropland and forest land-use types, respectively. In particular, FLNF increased sharply once soils crossed a critical moisture range, rising by more than two orders of magnitude at 60% WHC in cropland soil and 80% WHC in forest soil relative to drier treatments. This surge coincided with declining respiration at the wetter end of the gradient, consistent with the hypothesis that reduced oxygen inhibition of nitrogenase contributed to enhanced FLNF under high moisture. In addition, moisture-driven increases in FLNF were coordinated with pronounced shifts in diazotrophic community composition at both DNA- and RNA-based levels. In particular, *Azotobacter* (cropland) and *Paenibacillus* (forest) were positively associated with elevated FLNF, indicating that moisture might have filtered for taxa that were more abundant and active under wetter conditions. Overall, our study identifies pronounced moisture sensitivity of potential FLNF under controlled, C-replete incubation conditions and generates testable hypotheses concerning the possible roles of soil aeration and diazotrophic community composition. Validation across multiple independent soils and sites, together with direct measurements of O_2_ conditions, resource dynamics, absolute diazotroph abundance, and *in situ* N fixation, will be required before these findings can be generalized to ecosystem-scale N inputs or diazotroph-based biofertilizer applications.

## Data Availability

Raw nifH gene sequencing data have been deposited in Genome Sequence Archive database (GSA; https://ngdc.cncb.ac.cn/gsa/) under the accession number CRA025414.
