# Supplementary material for: Concomitant responses of free-living nitrogen fixation and diazotrophic community composition to soil moisture in two soils
Source: Front Microbiol. 2026 Aug 26;17:1915701. doi: 10.3389/fmicb.2026.1915701 (PMC13553789; doi:10.3389/fmicb.2026.1915701)
Supplement: Supplementary file 1 [file Table_1.docx]

Supplementary Material

# 1 Supplementary Figures and Tables

## 1.1 Supplementary Figures

**Supplementary Figure 1.** Effects of soil moisture (%water holding capacity, WHC) on relative abundances of dominant diazotrophic genera (DNA level) in the cropland soil on Day 3 (n = 4).

**Supplementary Figure 2.** Effects of soil moisture (%water holding capacity, WHC) on relative abundances of dominant diazotrophic genera (RNA level) in the cropland soil on Day 3 (n = 4).

**Supplementary Figure 3.** Effects of soil moisture (%water holding capacity, WHC) on relative abundances of dominant diazotrophic genera (DNA level) in the forest soil on Day 3 (n = 4).

**Supplementary Figure 4.** Effects of soil moisture (%water holding capacity, WHC) on relative abundances of dominant diazotrophic genera (RNA level) in the forest soil on Day 3 (n = 4).

## 1.2 Supplementary Tables

**Supplementary Table 1.** Basic properties and nutrient contents of the cropland and forest soils used in this study

| Land use type | pH | Bulk density | SOC | TN | EIN | EIP | SWC | WHC |
| --- | --- | --- | --- | --- | --- | --- | --- | --- |
|  |  | g cm^-3^ | mg g^-1^ | mg g^-1^ | mg kg^-1^ | mg kg^-1^ | % | % |
| Cropland | 8.22 | 1.32 | 26.2 | 0.11 | 22.1 | 3.16 | 19.6 | 52.3 |
| Forest | 8.19 | 0.93 | 53.3 | 0.25 | 1.4 | 2.15 | 15.2 | 58.0 |

Data are presented as the mean values of three replicate measurements.

SOC: soil organic carbon, TN: total N, EIN: the sum of soil NH_4_^+^-N and NO_3_^-^-N extracted by 2-M KCl, EIP: inorganic P extracted by 0.5-M NaHCO_3_, SWC: soil water content, WHC: water holding capacity.

**Supplementary Table 2.** Effects of soil moisture, sampling time, and their interactions on FLNF and respiration rates (4 replicates for each soil moisture × sampling time block)

| **Response variable** | **Factor** | **Wald_F** | ***P*** |
| --- | --- | --- | --- |
| FLNF rate (Cropland) | Soil moisture | 938.6 | < 0.001 |
|  | Sampling time | 236.7 | < 0.001 |
|  | Soil moisture × Sampling time | 131.7 | < 0.001 |
| FLNF rate (Forest) | Soil moisture | 1062 | < 0.001 |
|  | Sampling time | 189.9 | < 0.001 |
|  | Soil moisture × Sampling time | 94.76 | < 0.001 |
| Respiration rate (Cropland) | Soil moisture | 1.756 | = 0.150 |
|  | Sampling time | 126.0 | < 0.001 |
|  | Soil moisture × Sampling time | 130.5 | < 0.001 |
| Respiration rate (Forest) | Soil moisture | 10.34 | < 0.001 |
|  | Sampling time | 176.2 | < 0.001 |
|  | Soil moisture × Sampling time | 219.9 | < 0.001 |

**Supplementary** **Table 3.** Simple effects of soil moisture and sampling time on FLNF and respiration rates (4 replicates for each soil moisture × sampling time block)

| **Response variable** | **Tested factor** | **Level of interacting factor** | **F** | ***P*** |
| --- | --- | --- | --- | --- |
| FLNF rate (Cropland) | Soil moisture | Sampling time_Day1 | 109.1 | < 0.001 |
|  |  | Sampling time_Day3 | 614.5 | < 0.001 |
|  |  | Sampling time_Day5 | 5289 | < 0.001 |
|  |  | Sampling time_Day7 | 168.6 | < 0.001 |
|  |  | Sampling time_Day10 | 835.9 | < 0.001 |
|  |  | Sampling time_Day14 | 28.54 | < 0.001 |
|  | Sampling time | Soil moisture_20% | 5.225 | < 0.001 |
|  |  | Soil moisture_40% | 73.01 | < 0.001 |
|  |  | Soil moisture_60% | 5881 | < 0.001 |
|  |  | Soil moisture_80% | 122.0 | < 0.001 |
|  |  | Soil moisture_100% | 254.9 | < 0.001 |
| FLNF rate (Forest) | Soil moisture | Sampling time_Day1 | 75.07 | < 0.001 |
|  |  | Sampling time_Day3 | 283.4 | < 0.001 |
|  |  | Sampling time_Day5 | 377.4 | < 0.001 |
|  |  | Sampling time_Day7 | 426.9 | < 0.001 |
|  |  | Sampling time_Day10 | 318.7 | < 0.001 |
|  |  | Sampling time_Day14 | 21.54 | < 0.001 |
|  | Sampling time | Soil moisture_20% | 10.52 | < 0.001 |
|  |  | Soil moisture_40% | 5.662 | < 0.001 |
|  |  | Soil moisture_60% | 3.279 | 0.009 |
|  |  | Soil moisture_80% | 279.9 | < 0.001 |
|  |  | Soil moisture_100% | 1677 | < 0.001 |
| Respiration rate (Cropland) | Soil moisture | Sampling time_Day1 | 1039 | < 0.001 |
|  |  | Sampling time_Day3 | 153.8 | < 0.001 |
|  |  | Sampling time_Day5 | 4.486 | = 0.003 |
|  |  | Sampling time_Day7 | 1.513 | = 0.210 |
|  |  | Sampling time_Day10 | 0.2092 | = 0.932 |
|  |  | Sampling time_Day14 | 0.2075 | = 0.872 |
|  | Sampling time | Soil moisture_20% | 274.9 | < 0.001 |
|  |  | Soil moisture_40% | 751.0 | < 0.001 |
|  |  | Soil moisture_60% | 174.0 | < 0.001 |
|  |  | Soil moisture_80% | 15.75 | < 0.001 |
|  |  | Soil moisture_100% | 15.35 | < 0.001 |
| Respiration rate (Forest) | Soil moisture | Sampling time_Day1 | 770.3 | < 0.001 |
|  |  | Sampling time_Day3 | 416.2 | < 0.001 |
|  |  | Sampling time_Day5 | 4.205 | = 0.005 |
|  |  | Sampling time_Day7 | 2.851 | = 0.031 |
|  |  | Sampling time_Day10 | 1.451 | = 0.228 |
|  |  | Sampling time_Day14 | 7.415 | < 0.001 |
|  | Sampling time | Soil moisture_20% | 1330 | < 0.001 |
|  |  | Soil moisture_40% | 2512 | < 0.001 |
|  |  | Soil moisture_60% | 645.1 | < 0.001 |
|  |  | Soil moisture_80% | 52.22 | < 0.001 |
|  |  | Soil moisture_100% | 25.09 | < 0.001 |

**Supplementary** **Table 4.** Pairwise comparison of FLNF rate and respiration rate among different levels of soil moisture and different sampling times with significant levels adjusted using the Holm procedure (4 replicates for each soil moisture × sampling time block)

| **FLNF rate (cropland)** | **Day 1** | **Day 3** | **Day 5** | **Day 7** | **Day 10** | **Day 14** |
| --- | --- | --- | --- | --- | --- | --- |
| 20% WHC | 0.003±0.002 eA | 0.002±0.001 dA | 0.003±0.001 cA | 0.002±0.002 cAB | 0.001±0.001 dAB | 0.001±0.001 bB |
| 40% WHC | 0.030±0.005 dA | 0.022±0.010 cA | 0.003±0.001 cB | 0.001±0.001 cBC | 0.003±0.001 cB | 0.001±0.001 bC |
| 60% WHC | 0.089±0.018 cC | 4.66±0.566 bB | 9.75±2.80 bA | 13.9±3.80 bA | 11.8±0.570 aA | 0.009±0.001 aD |
| 80% WHC | 0.180±0.075 bC | 14.9±3.73 aB | 39.6±8.31 aA | 32.5±15.1 abAB | 0.105±0.154 bCD | 0.019±0.012 aD |
| 100% WHC | 0.628±0.157 aC | 15.2±1.27 aB | 30.7±0.743 aA | 28.3±2.33 aA | 0.293±0.225 bC | 0.028±0.026 aD |
| **FLNF rate (forest)** | **Day 1** | **Day 3** | **Day 5** | **Day 7** | **Day 10** | **Day 14** |
| 20% WHC | 0.005±0.001 bA | 0.002±0.001 cB | 0.002±0.001 eB | 0.002±0.003 dABC | 0.003±0.002 cABC | 0.001±0.001 cC |
| 40% WHC | 0.007±0.003 bA | 0.004±0.002 cAB | 0.006±0.001 dA | 0.005±0.003 dAB | 0.006±0.002 cAB | 0.003±0.001 bB |
| 60% WHC | 0.008±0.003 bAB | 0.059±0.055 bAB | 0.008±0.001 cAB | 0.019±0.013 cA | 0.007±0.003 cAB | 0.004±0.002 abB |
| 80% WHC | 0.261±0.178 aB | 3.57±1.76 aA | 2.87±1.31 bA | 4.83±1.35 bA | 4.83±1.82 bA | 0.005±0.001 aC |
| 100% WHC | 0.783±0.414 aD | 2.14±0.518 aC | 10.4±3.28 aB | 17.7±1.30 aA | 12.4±4.60 aAB | 0.007±0.001 aE |
| **Respiration rate (cropland)** | **Day 1** | **Day 3** | **Day 5** | **Day 7** | **Day 10** | **Day 14** |
| 20% WHC | 396±13.4 dB | 1280±51.6 aA | 211±30.0 aC | 174±67.3 aC | 65.4±13.1 aD | 93.6±52.8 aCD |
| 40% WHC | 1780±11.4 aA | 431±24.4 bB | 138±15.6 bC | 101±16.7 aCD | 74.0±10.9 aD | 63.4±43.5 aCD |
| 60% WHC | 1110±49.9 bA | 300±167 bAB | 148±29.6 abB | 116±14.9 aB | 66.9±21.0 aB | 41.2±25.8 aB |
| 80% WHC | 708±186 cA | 395±50.9 bAB | 196±105 abBC | 157±39.7 aC | 82.2±34.2 aC | 60.9±29.2 aC |
| 100% WHC | 342±92.4 dA | 326±67.3 bA | 208±24.5 aAB | 131±57.6 aABC | 75.6±14.0 aC | 66.1±47.5 aBC |
| **Respiration rate (forest)** | **Day 1** | **Day 3** | **Day 5** | **Day 7** | **Day 10** | **Day 14** |
| 20% WHC | 591±16.8 bB | 1560±40.0 aA | 249±40.0 abC | 50.2±35.6 aE | 90.9±14.0 aE | 153±5.00 bD |
| 40% WHC | 1940±25.3 aA | 596±16.0 bB | 256±9.60 bC | 116±27.4 aE | 98.6±23.3 aE | 177±7.00 aD |
| 60% WHC | 1970±57.6 aA | 441±70.3 cB | 213±34.7 bC | 154±9.90 aC | 130±26.2 aC | 178±36.7 abcC |
| 80% WHC | 806±148 bA | 145±23.2 dC | 369±47.9 aB | 159±20.3 aC | 117±12.5 aC | 115±16.1 cC |
| 100% WHC | 506±227 bAB | 549±89.6 bcA | 322±145 abABC | 228±90.8 aABC | 115±12.9 aC | 140±75.1 abcBC |

Note: lowercase letters indicate significant differences among moisture levels within each sampling time, while uppercase letters indicated significant differences among sampling times within each moisture level.

**Supplementary Table 5.** Model comparison for selection of the best model fitting the relationships between soil moisture and cumulative FLNF rate (4 replicates for each soil moisture × sampling time block)

| Cropland model | Parameter number | LOOCV RMSE | LOOCV asinh ratio | Selection score |
| --- | --- | --- | --- | --- |
| Linear | 2 | 55.04 | 0.4706 | 0.6594±0.1698 |
| **Logistic** | **3** | **18.47** | **0.1579** | **0.1807±0.0239** |
| Logarithmic | 2 | 71.99 | 0.6155 | 0.8223±0.2769 |
| Quadratic | 3 | 128.0 | 1.095 | 1.123±0.2898 |
| Gaussian peak | 3 | 482.8 | 4.128 | 2.590±0.6275 |
| Power | 2 | 795.6 | 6.802 | 2.702±1.120 |
| Exponential | 2 | 15040 | 128.6 | 46.24±20.26 |
| Forest model | Parameter number | LOOCV RMSE | LOOCV asinh ratio | Selection score |
| Linear | 2 | 45.58 | 0.7366 | 0.8034±0.1592 |
| Logistic | 3 | 37.77 | 0.1763 | 0.4484±0.1132 |
| Logarithmic | 2 | 64.14 | 0.8583 | 0.9808±0.2080 |
| Quadratic | 3 | 18.60 | 0.6427 | 0.7055±0.1483 |
| Gaussian peak | 3 | 51.48 | 0.5998 | 1.809±0.2918 |
| **Power** | **2** | **35.06** | **0.3114** | **0.4194±0.1105** |
| Exponential | 2 | 43.95 | 0.2270 | 0.4195±0.1279 |

**Note**: the selected model was denoted in bold. Model performance was assessed using leave-one-moisture-level-out cross-validation (LOOCV). Prediction performance was evaluated using both raw-scale RMSE and RMSE calculated after inverse hyperbolic sine transformation (asinh), which allows robust evaluation of zero and slightly negative predictions. Errors were normalized against a null model to quantify the relative improvement of each candidate model. The final model selection score combined normalized prediction errors from both scales, negative prediction penalties, prediction instability penalties, shape constraints for peak models, and model complexity penalties. A one-standard-error rule was applied to avoid selecting unnecessarily complex models when multiple models showed comparable predictive performance.

**Abbreviation**: *Parameters number*, indicates model complexity; a smaller represents a more parsimonious model when predictive performances are comparable. *LOOCV RMSE*, Leave-One-Out Cross-Validation Root Mean Square Error, measures the average magnitude of cross-validated prediction errors and gives greater weight to large deviations; lower values indicate better predictive accuracy. *LOOCV asinh ratio*, Leave-One-Out Cross-Validation Inverse Hyperbolic Sine-Scale Error Ratio, is the cross-validated prediction error on the inverse hyperbolic sine-transformed scale relative to that of the null model; values below 1 indicate better performance than the null model, and smaller values indicate stronger predictive improvement. *Selection score*, integrates cross-validated prediction errors, prediction penalties and model-complexity penalties; lower values indicate better overall performance, stability and parsimony.

**Supplementary Table 6.** Correlations between FLNF rate and the two ordination axes in the PCoA analysis as shown in Fig. 3 of the main text (4 replicates for each soil moisture level, Day 3)

| Level | Soil | *r*_PCoA1 | *r_*PCoA2 | R^2^ | *P* | Corresponding figure in the main text |
| --- | --- | --- | --- | --- | --- | --- |
| DNA | Cropland | 0.72 | −0.69 | 0.92 | **< 0.001** | Fig. 3a |
|  | Forest | 0.98 | −0.19 | 0.65 | **0.003** | Fig. 3b |
| RNA | Cropland | 0.44 | −0.90 | 0.52 | **0.003** | Fig. 3c |
|  | Forest | 0.86 | −0.51 | 0.66 | **< 0.001** | Fig. 3d |

*r*_PCoA1 and *r*_PCoA2: correlation efficient between FLNF rate and the first and second ordination axes, respectively. The R^2^ and P values are used to represent the relationship between the FLNF rate and diazotrophic community. A *P* value of < 0.05 indicates significant correlation, and the larger the R^2^ value (ranging from 0 to 1), the stronger the correlation. The permutation tests were based on 999 permutations to assess the significance of the relationships.

**Supplementary Table 7.** Correlations between FLNF rate and the top-9 diazotrophic genera on DNA and RNA level on (4 replicates for each soil moisture level, Day 3)

| Soil | *level* | *genera* | *r* | *P_adj_* |
| --- | --- | --- | --- | --- |
| Cropland | DNA | ***Azotobacter*** | 0.93 | **<0.001** |
|  |  | *Paenibacillus* | 0.52 | 0.185 |
|  |  | *Skermanella* | −0.88 | **<0.001** |
|  |  | *g__unclassified_p__Proteobacteria* | −0.88 | **<0.001** |
|  |  | *g__unclassified_k__norank_d__Bacteria* | −0.86 | **<0.001** |
|  |  | *g__unclassified_c__Alphaproteobacteria* | −0.82 | **<0.001** |
|  |  | *Azohydromonas* | −0.71 | **0.005** |
|  |  | *g__unclassified_f__Rhodospirillaceae* | −0.74 | **0.002** |
|  |  | *Nostoc* | −0.81 | **<0.001** |
|  |  | *g__unclassified_f__Nostocaceae* | −0.74 | **0.002** |
|  | RNA | ***Azotobacter*** | 0.46 | **0.043** |
|  |  | *Paenibacillus* | 0.28 | 1.000 |
|  |  | *Nostoc* | −0.40 | 0.082 |
|  |  | *g__unclassified_k__norank_d__Bacteria* | −0.34 | 1.000 |
|  |  | *Skermanella* | −0.42 | 0.062 |
|  |  | *g__unclassified_f__Nostocaceae* | −0.38 | 0.097 |
|  |  | *Anabaena* | −0.25 | 1.000 |
|  |  | *g__unclassified_p__Proteobacteria* | −0.30 | 1.000 |
|  |  | *g__unclassified_o__Nostocales* | −0.34 | 1.000 |
|  |  | *g__unclassified_o__Burkholderiales* | 0.25 | 1.000 |
| Forest | DNA | *g__unclassified_k__norank_d__Bacteria* | 0.48 | 0.327 |
|  |  | ***Paenibacillus*** | 0.78 | **<0.001** |
|  |  | *g__unclassified_p__Proteobacteria* | −0.65 | **0.018** |
|  |  | *Skermanella* | −0.73 | **0.003** |
|  |  | *Bradyrhizobium* | −0.75 | **0.001** |
|  |  | *Azotobacter* | −0.60 | **0.047** |
|  |  | *g__unclassified_c__Alphaproteobacteria* | −0.75 | **0.001** |
|  |  | *Sinorhizobium* | −0.58 | 0.072 |
|  |  | *g__unclassified_f__Beijerinckiaceae* | −0.65 | **0.018** |
|  |  | *g__unclassified_f__Rhodospirillaceae* | −0.62 | **0.038** |
|  | RNA | ***Paenibacillus*** | 0.699 | **0.006** |
|  |  | *g__unclassified_k__norank_d__Bacteria* | 0.458 | 0.425 |
|  |  | *g__unclassified_p__Proteobacteria* | −0.342 | 1.000 |
|  |  | *Bradyrhizobium* | −0.751 | **0.001** |
|  |  | *Skermanella* | −0.676 | **0.011** |
|  |  | *Azotobacter* | −0.367 | 1.000 |
|  |  | *g__unclassified_c__Alphaproteobacteria* | −0.649 | **0.020** |
|  |  | *Sinorhizobium* | −0.385 | 0.937 |
|  |  | *g__unclassified_f__Beijerinckiaceae* | −0.688 | **0.008** |
|  |  | *g__unclassified_f__Rhodospirillaceae* | −0.554 | **0.011** |

Relationships between the abundances of the top-10 most abundant species and the FLNF rate were evaluated using Pearson correlation tests. Bonferroni correction was applied to adjust the *P*-values for multiple comparisons. A *P* value of < 0.05 indicates significant correlation. Significant *P_adj_* values are highlighted in bold.
